# Establishment of blood glycosidase activities and their excursions in sepsis

**DOI:** 10.1093/pnasnexus/pgac113

**Published:** 2022-07-11

**Authors:** Benjamin S Haslund-Gourley, Peter V Aziz, Douglas M Heithoff, Damien Restagno, Jeffrey C Fried, Mai-Britt Ilse, Hannah Bäumges, Michael J Mahan, Torben Lübke, Jamey D Marth

**Affiliations:** Sanford-Burnham-Prebys Medical Discovery Institute, Infectious and Inflammatory Diseases Center, La Jolla, CA 92037, USA; Sanford-Burnham-Prebys Medical Discovery Institute, Infectious and Inflammatory Diseases Center, La Jolla, CA 92037, USA; Department of Molecular, Cellular, and Developmental Biology, University of California Santa Barbara, CA 93106, USA; Sanford-Burnham-Prebys Medical Discovery Institute, Infectious and Inflammatory Diseases Center, La Jolla, CA 92037, USA; Department of Pulmonary and Critical Care Medicine, Cottage Hospital of Santa Barbara, Santa Barbara, CA 93105, USA; Department of Chemistry, Biochemistry, Bielefeld University, D-33615, Germany; Department of Chemistry, Biochemistry, Bielefeld University, D-33615, Germany; Department of Molecular, Cellular, and Developmental Biology, University of California Santa Barbara, CA 93106, USA; Department of Chemistry, Biochemistry, Bielefeld University, D-33615, Germany; Sanford-Burnham-Prebys Medical Discovery Institute, Infectious and Inflammatory Diseases Center, La Jolla, CA 92037, USA

## Abstract

Glycosidases are hydrolytic enzymes studied principally in the context of intracellular catabolism within the lysosome. Therefore, glycosidase activities are classically measured in experimentally acidified assay conditions reflecting their low pH optima. However, glycosidases are also present in the bloodstream where they may retain sufficient activity to participate in the regulation of glycoprotein half-lives, proteostasis, and disease pathogenesis. We have, herein, established at physiological pH 7.4 in blood plasma and sera the normal ranges of four major glycosidase activities essential for blood glycoprotein remodeling in healthy mice and humans. These activities included β-galactosidase, β-N-acetylglucosaminidase, α-mannosidase, and α-fucosidase. We have identified their origins to include the mammalian genes *Glb1, HexB, Man2a1*, and *Fuca1*. In experimental sepsis, excursions of glycosidase activities occurred with differences in host responses to discrete bacterial pathogens. Among similar excursions in human sepsis, the elevation of β-galactosidase activity was a prognostic indicator of increased likelihood of patient death.

Significance Statement Blood levels of glycosidase enzymes have been previously studied in experimentally acidified low pH conditions including in the detection of various human diseases. However, the likely presence of bioactive glycosidase activity at physiological pH 7.4 has not been fully investigated in the blood. We have, herein, established the normal activity ranges of four major glycosidase activities in the blood plasma and sera of mice and humans, demonstrating significant glycosidase activity remains present to function at physiological pH 7.4 in the bloodstream. We have identified the genes contributing to these activities and recorded significant excursions of blood glycosidase activities outside of normal ranges in sepsis. One such finding further identified a novel prognostic indicator of increased risk of patient death.

## Introduction

Glycosidases are catabolic enzymes that hydrolyze glycan linkages from glycoproteins and other glycoconjugates (1, 2). Numerous glycosidases are encoded within mammalian genomes, and each enzyme typically cleaves a distinct anomeric glycosidic linkage produced during the process of glycosylation in the endoplasmic reticulum and Golgi apparatus. Most are exoglycosidases that hydrolyze terminal (nonreducing end) glycosidic linkages of polymeric glycan structures in a stepwise manner in reverse order of glycan synthesis. They include amongst others α-neuraminidase, β-galactosidase, β-N-acetylglucosaminidase, α-mannosidase, and α-fucosidase.

Inherited mutations in glycosidase genes are the origins of several human lysosomal storage diseases including sialidosis due to deficiency of neuraminidase *NEU1* (3, 4), GM1 gangliosidosis and Morquio B disease due to deficiency of β-galactosidase GLB1 (5, 6), Sandhoff disease/GM2 gangliosidosis due to deficiency of β-N-acetylglucosaminidase *HEXB* (7), mannosidosis due to deficiency of α-mannosidase *MAN2B1* (8, 9), and fucosidosis due to deficiency of tissue α-L-fucosidase *FUCA1* (10). The pathobiology of these disorders has been ascribed to catabolic failure resulting in the accumulation of glycosidase substrates leading to cell dysfunction and death. Disease diagnosis has included comparative measurements of glycosidase activity levels present in experimentally acidified blood plasma and sera reflecting the low pH optima (4.3 to 5.5) of these enzymes to achieve optimal assay sensitivity (11–13).

Glycosidases at physiological pH 7.4 in blood plasma and sera may retain significant activity to operate in normal and pathogenic glycan remodeling of secreted and cell surface glycoproteins. Indeed, we found previously that neuraminidase activity in the blood alters glycoprotein half-lives in contributing to the pathogenesis of sepsis (4, 5). Circulating neuraminidase (Neu) activity has been relatively well-studied with other roles in inflammatory and immune diseases in mice and humans (16–20). However, much less is known of the subsequent glycolytic steps involving β-galactosidase, β-N-acetylglucosaminidase, α-mannosidase, and α-fucosidase activities. Using mouse and human blood samples, we herein, establish the normal ranges of these four major glycosidase activities in the blood plasma and sera of males and females at physiological pH 7.4, and identify the specific glycosidases contributing these activities. Our studies further reveal excursions of blood glycosidase activities in the context of sepsis with elevated β-galactosidase activity in the plasma of human sepsis patients predictive of a significantly increased risk of death.

## Results

### β-galactosidase activity and origin in blood plasma and serum

Assays of all four glycosidase activities used 4-methylumbelliferyl (4-MU) substrate analogs. Background fluorescence of the parental uncleaved conjugate limited sensitivity to 1 to 2 mU/L in both assay time courses, nevertheless specifc activity measurements were achieved spanning three-four orders of magnitude. β-galactosidase activity cleaves the beta anomeric glycosidic linkage of galactose from glycoconjugates. The β-galactose linkage is commonly present in multiple glycoconjugates including the asparagine-linked (N-) and serine/threonine-linked (O-) glycans of vertebrates (21). β-galactose is typically the penultimate glycosidic linkage underlying sialic acid linkages at the terminal nonreducing end among mature nascent glycoproteins.

**Fig. 1.**
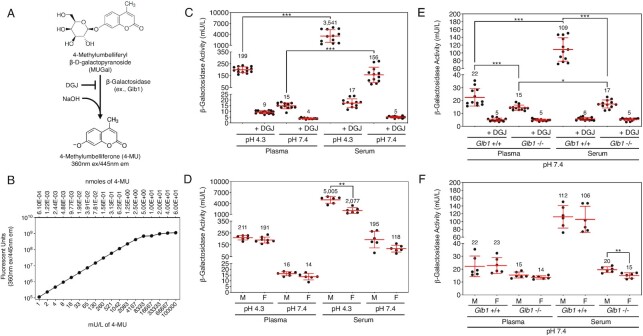
β-galactosidase activity and genetic source(s) in murine blood plasma and sera. (A) β-galactosidase assay used to determine specific activity at pH 7.4 compared to pH 4.3 with and without the addition of the inhibitor N-(n-nonyl)-DGJ (C*_f_* = 1 mM). (B) Standard curve of fluorescence measured from serial dilutions of the fluorescent reporter 4-methylumbelliferone (4-MU) used to convert 4-MU fluorescence into units of enzyme activity at 60 min following assay start. A unit was defined as 1 μmole of substrate hydrolyzed per minute. (C) β-galactosidase activity in plasma and serum from wild-type mice (*n* = 14). (D) β-galactosidase activity in the plasma and serum of wild-type male and female littermates (*n* = 7 per sex). (E) β-galactosidase activity in plasma and serum of *Glb1*-null mice compared to wild-type littermates at pH 7.4 (*n* = 12 per genotype). (F) β-galactosidase activity in the plasma and serum of *Glb1*-null male and female mice at pH 7.4 compared to wild-type littermates (*n* = 6 per sex). Data are presented as means ± SD. Unpaired Mann–Whitney nonparametric statistical significance is denoted by *P < 0.05, **P < 0.01, or ***P < 0.001.

Measurements of blood β-galactosidase activity were acquired from platelet-poor plasma and sera of wild-type C57BL/6 J mice at the pH 4.3 optimum of β-galactosidase activity and at physiological pH 7.4 in assays using the substrate 4-methylumbelliferyl-β-D-galactopyranoside and the galactosidase inhibitor N-(n-nonyl)-deoxygalactonojirimycin (DGJ; Figure 1A) (22). The fluorescence of standardized 4-MU concentrations was used in calculating specific activity among plasma and sera samples (Figure 1B). Compared to pH 7.4, acidification of plasma and sera to pH 4.3 resulted as expected in high levels of activity. However, 4% to 8% of β-galactosidase activity measured at pH 4.3 remained in the plasma and sera when analyzed at pH 7.4 (Figure 1C). Remarkably, β-galactosidase activity in the sera was increased by 10- to 17-fold compared to plasma, regardless of pH, with activity levels in the sera at pH 7.4 matching activity in the acidified platelet-poor plasma. This implicates platelets as a source of the majority of β-galactosidase activity measured among sera samples compared with platelet-poor plasma. In comparing males and females, sera from females contained lower levels of β-galactosidase activity (Figure1D). DGJ inhibition results included the presence of small levels of residual activity that may represent other isozymes (see below).

Deficient β-galactosidase activity in humans is linked to mutations of the *GLB1* allele in the autosomal recessive syndromes GM1 gangliosidosis and Morquio B disease (5, 23). The *Glb1*-null mouse model does not present overt disease phenotypes until several months into adult life (24). At early nonsymptomatic adult ages, we measured β-galactosidase activity in the plasma and sera of *Glb1*-null mice at pH 7.4 and compared the results to wild-type littermates. β-galactosidase activity was not completely eliminated and remained higher in the sera compared to plasma in GLB1 deficiency, implying the presence of an isozyme, perhaps β-galactosylceramidase, contributing a low level of activity in this assay (Figure 1E and F).

### β-N-acetylglucosaminidase activity and origin in blood plasma and serum

**Fig. 2.**
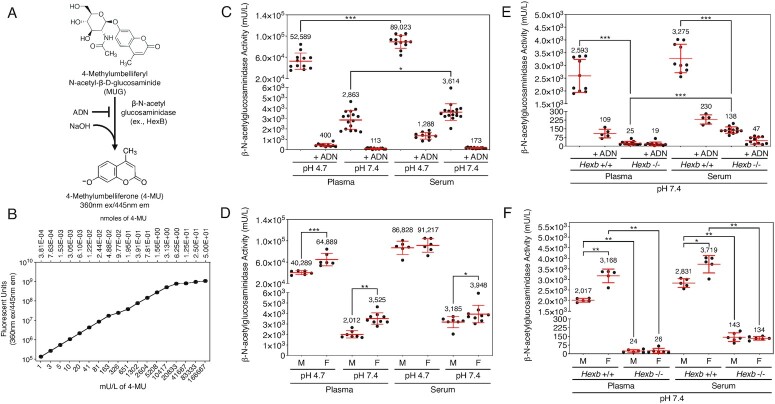
β-N-acetylglucosaminidase activity and genetic source(s) in murine blood plasma and sera. (A) β-N-acetylglucosaminidase assay used to determine specific activity at pH 7.4 compared to pH 4.7 with and without the addition of the inhibitor ADN (C*_f_* = 0.25 mM). (B) Standard curve of fluorescence measured from serial dilutions of the enzyme product and fluorescent reporter 4-MU used to convert 4-MU fluorescence into units of enzyme activity at 60 min following assay start. A unit was defined as 1 μmole of substrate hydrolyzed per min. (C) β-N-acetylglucosaminidase activity in plasma and serum from wild-type mice (*n* = 12). (D) β-N-acetylglucosaminidase activity in the plasma and serum of wild-type male and female littermates (*n* = 6 per sex). (E) β-N-acetylglucosaminidase activity in plasma and serum of *HexB*-null mice compared to wild-type littermates at pH 7.4 (*n* = 10 per genotype). (F) β-N-acetylglucosaminidase activity in the plasma and serum of male and female *HexB*-null mice at pH 7.4 compared to wild-type littermates (*n* = 5 per sex). Assay background was 1 mU/L or less. Data are presented as means ± SD. Unpaired Mann–Whitney nonparametric statistical significance is denoted by *P < 0.05, **P < 0.01, or ***P < 0.001.

β-N-acetylglucosaminidase cleaves the N-acetylglucosamine linkage from glycoconjugates. During glycan degradation in the lysosome, exoglycosidic hydrolysis of the β-N-acetylglucosamine linkage follows elimination of the β-galactose linkage by β-galactosidase activity. β-N-acetylglucosaminidase activity has also been detected in acidified blood samples (14). We assayed β-N-acetylglucosaminidase activity in plasma and sera using cleavage of the substrate 4-methylumbelliferyl-N-acetyl-β-D-glucosamide in the presence and absence of the β-N-acetylglucosaminidase inhibitor 2-acetamido-1,2-dideoxynojirimycin (ADN) that inhibits HexA and HexB isozymes (Figure 2A) (25). Units of β-N-acetylglucosaminidase activity were calculated based on the fluorescence of standardized 4-MU concentrations (Figure 2B).

β-N-acetylglucosaminidase activity measurements were acquired from acidified platelet-poor plasma and sera of wild-type C57BL/6 J mice at the pH 4.7 optimum and compared to findings at pH 7.4. As expected, the acidification of plasma and sera resulted in high levels of β-N-acetylglucosaminidase activity. On average, 5% of β-N-acetylglucosaminidase activity remained in the plasma and sera at pH 7.4 (Figure 2C). β-N-acetylglucosaminidase activity was also significantly higher in sera compared to plasma samples (Figure 2C), indicating release from platelets during sera preparation. Comparing males and females, β-N-acetylglucosaminidase activity was significantly increased among female plasma samples at either pH as well as in the sera at pH 7.4 (Figure 2D). ADN inhibited over 90% of activity in these assays, however, residual activity remained which may indicate other isozymes (see below).

Human deficiency of the β-N-acetylglucosaminidase HEXB results in the autosomal recessive lysosomal storage disorder known as Sandhoff disease with neural accumulation of GM2 gangliosides (7). *HexB*-null mice have been studied as a model of this disease and appear healthy during the first 3 to 4 months of adult life prior to the onset of symptoms (26, 27). Plasma and sera samples were obtained from young adult *HexB*-null mice and assayed at pH 7.4. β-N-acetylglucosaminidase activity was reduced by over 99% in the plasma of *HexB*-null littermates while 4% activity remained in the sera (Figure 2E). HexB deficiency reduced β-N-acetylglucosaminidase activity similarly in the plasma and sera of males and females (Figure 2F).

### α-Mannosidase activity and origin in mouse plasma and serum

**Fig. 3.**
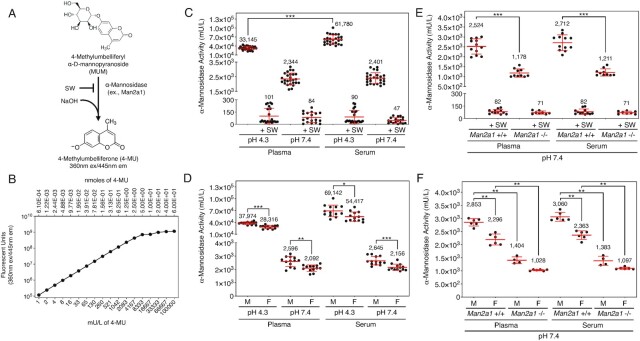
α-Mannosidase activity and genetic source(s) in murine blood plasma and sera. (A) α-Mannosidase assay used to determine specific activity at pH 7.4 compared to pH 4.3 with and without the addition of the inhibitor SW (C*_f_* = 0.25 mM). (B) Standard curve of fluorescence measured from serial dilutions of the enzyme product and fluorescent reporter 4-MU used to convert 4-MU fluorescence into units of enzyme activity at 60 min following assay start. A unit was defined as 1 μmole of substrate hydrolyzed per minute. (C) α-Mannosidase activity in plasma and serum of wild-type mice (*n* = 26). (D) α-Mannosidase activity in the plasma and serum of wild-type male and female littermates (*n* = 13 per sex). (E) α-Mannosidase activity in plasma and serum of *Man2a1*-null mice compared to wild-type littermates at pH 7.4 (*n* = 12 per genotype). (F) α-Mannosidase activity in plasma and serum of male and female *Man2a1*-null mice at pH7.4 compared to wild-type littermates (*n* = 6 per sex). Data are presented as means ± SD. Unpaired Mann–Whitney nonparametric statistical significance is denoted by *P < 0.05, **P < 0.01, or ***P < 0.001.

α-Mannosidase activity participates in glycan biosynthetic pathways in the endoplasmic reticulum and the Golgi and in the catabolism of mannosylated glycan linkages in the lysosome. Mostly found in N-glycans, mannose linkages are sequentially processed during N-glycan synthesis and then used as platform substrates for N-glycan branching initiated by N-acetylglucosamine linkages added to the tri-mannosyl core structure. We measured α-mannosidase activity by cleavage of the substrate 4-methylumbelliferyl-α-D-mannopyranoside in the presence and absence of the α-mannosidase II activity inhibitor swainsonine (SW; Figure 3A) (28). Units of activity were calculated based upon the fluorescence of 4-MU at standardized concentrations (Figure 3B).

α-Mannosidase activity measurements were acquired from platelet-poor plasma and sera of wild-type C57BL/6 J mice at pH 4.3 compared to pH 7.4. Acidification of plasma and sera to pH 4.3 resulted in highest levels of α-mannosidase activity again compared to pH 7.4. Nevertheless, on average 7% of specific activity remained at pH 7.4 (Figure 3C). In addition, α-mannosidase activity was increased by almost 2-fold in sera compared to plasma at pH 4.3, but not at pH.7.4, which may reflect platelet release of two or more isozymes with different pH optima in the sera (Figure 3C). Comparing males and females, α-mannosidase activity was significantly decreased by 20% in both the plasma and sera of female littermates regardless of pH (Figure 3D). Using SW, we obtained over 90% inhibition of α-mannosidase activity. Residual activity further indicates the possible contribution of other α-mannosidase isozymes.

Nine α-mannosidases are encoded in the mammalian genome and include the exoglycosidase α-mannosidase-II residing in the Golgi and produced by the *Man2a1* gene. Among other mannosidases, human MAN1B1 deficiency has been linked to intellectual disability and MAN2B1 deficiency in humans causes a lysosomal storage disease known as alpha-mannosidosis (29). In contrast, Man2a1 deficiency in mice is well-tolerated at early ages, however, these animals develop dyserythropoiesis and in later adult life autoimmune disease (30–32). Plasma and sera samples were obtained from young adult *Man2a1*-null mice and assayed at pH 7.4. Our findings revealed that Man2a1 contributes on average 50% of the α-mannosidase activity present in mouse plasma and sera (Figure 3E), indicating the presence of multiple α-mannosidases in circulation. Additional studies of Man2a1 deficiency revealed that females expressed 67% to 75% of α-mannosidase activity measured in males (Figure 3F).

### α-Fucosidase activity and origin in mouse plasma and serum

**Fig. 4.**
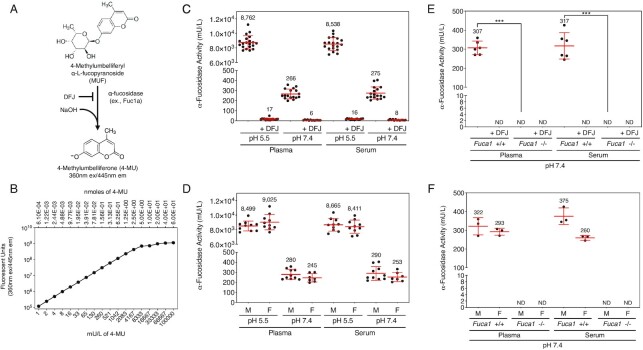
α-Fucosidase activity and genetic source(s) in murine blood plasma and sera. (A) α-Fucosidase assay used to determine specific activity at pH 7.4 compared to pH 5.5 with and without the addition of the inhibitor DFJ (C*_f_* = 0.2 mM). (B) Standard curve of fluorescence measured from serial dilutions of the enzyme product and fluorescent reporter 4-MU used to convert 4-MU fluorescence into units of enzyme activity at 60 min following assay start. A unit was defined as 1 μmole of substrate hydrolyzed per minute. (C) α-Fucosidase activity in plasma and serum from wild-type mice (*n* = 20). (D) α-Fucosidase activity in the plasma and serum of wild-type male and female littermates (*n* = 10 per sex). (E) α-Fucosidase activity in plasma and serum of *Fuca1*-null mice at pH 7.4 compared to wild-type littermates. (*n* = 6 per genotype). (F) α-Fucosidase activity in plasma and serum of male and female *Fuca1*-null mice at pH 7.4 compared to wild-type littermates (*n* = 3 per sex). ND; not detected. Data are presented as means ± SD. Unpaired Mann–Whitney nonparametric statistical significance is denoted by *P < 0.05, **P < 0.01, or ***P < 0.001.

Tissue α-L-Fucosidase activity cleaves α-fucose linkages from glycoconjugates during glycan catabolism in the lysosome (33). Like sialic acid, α-linked fucose is found at the terminal nonreducing end of various branched glycan polymers. α-fucosidase activity is also responsible for the hydrolysis of α-1,6-linked (core-) fucose of N-glycans, which explains the accumulation of α-1,6-fucosylated glycoasparagines in fucosidosis (34). We measured α-fucosidase activity by cleavage of the substrate 4-methylumbelliferyl-α-L-fucopyranoside in the presence and absence of the α-fucosidase inhibitor 1,5-dideoxy-1,5-imino-L-fucitol (deoxyfuconojirimycin, DFJ; Figure 4A) (35). Units of activity were calculated from 4-MU fluorescence acquired at standardized concentrations (Figure 4B).

α-Fucosidase activity measurements were acquired from platelet-poor plasma and sera of wild-type C57BL/6 J mice at the pH 5.5 optimum and compared to nonacidified assay conditions at pH 7.4. As expected, the acidification of plasma and sera resulted in high levels of α-fucosidase activity as compared with pH 7.4. Nevertheless, 3% of α-fucosidase activity remained at pH 7.4 as determined further in the presence of the inhibitor DFJ (Figure 4C). DFJ inhibited on average 97% of fucosidase activity, however, 3% of α-fucosidase activity remained at pH 7.4. No differences in activity were detected when comparing plasma and sera or when comparing males and females at either pH (Figure 4D). The addition of DFJ inhibited on average 97% of fucosidase activity.

Two fucosidase genes have been identified in the mammalian genome including *Fuca1* and *Fuca2* (36, 37). We measured α-fucosidase activity in the plasma and sera of *Fuca1*-null mice compared with wild-type littermates with and without the inhibitor DFJ. Absence of Fuca1 resulted in the loss of all measurable α-fucosidase activity in the plasma and sera with identical findings among males and females (Figure 4E and F). These results indicate a single fucosidase (Fuca1) contributes to activity in the blood, and further indicates that incomplete inhibition of α-fucosidase activity by DFJ among wild-type plasma and sera represents a limitation of inhibitor function in our assay.

These findings together have established the normal glycosidase activity ranges for β-galactosidase, β-N-acetylglucosaminidase, α-mannosidase, and α-fucosidase in the blood plasma and sera of laboratory mice (Table S1, Supplementary Material). This knowledge facilitates experimentation to determine whether changes in glycosidase activities are linked to diseases including sepsis.

### Blood plasma glycosidase activities during experimental bacterial sepsis

Bacterial sepsis was elicited and compared in wild-type C57BL/6 J mice separately using five bacterial pathogens known to cause human sepsis. These included *Salmonella enterica* Typhimurium (*ST*), *Escherichia coli* (*EC*), *Streptococcus pneumoniae* (*SP*), *Staphylococcus aureus* (*SA*), and Methicillin-resistant *Staphylococcus aureus* (*MR*). Bacterial inoculates representing 20X LD_50_ titers were administrated with the subsequent acquisition of two postinfection timepoints linked to escalating bacteremia (cfu) as described (15). This protocol included the acquisition of platelet poor plasma from early and late sepsis timepoints associated with the absence and presence of overt disease signs, respectively.

**Fig. 5.**
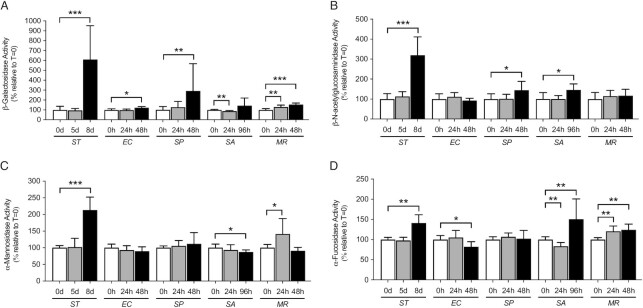
Glycosidase activities in mouse plasma at pH 7.4 during experimental sepsis. Mouse cohorts were infected with clinically isolated bacterial pathogens and analyzed for changes in plasma glycosidase activities at two times postinfection measured either in hours (h) or days (d) during the pathogenesis of sepsis compared to uninfected littermates at time 0 (*t* = 0). Activities relative to uninfected littermate cohorts were plotted and include (A) β-galactosidase activity (*n* = 8 per timepoint including four males and four females), which increased at later infection timepoints on average 600% in sepsis caused by *ST*, 120% by *EC*, 292% by *SP*, and 153% by *MR* infections, (B) β-N-acetylglucosaminidase activity (*n* = 8 per timepoint including four males and four females), which was increased on average 320% by *ST*, 145% by *SP*, and 146% by *SA* infections at the late sepsis timepoint, (C) α-mannosidase activity (*n* = 8 per timepoint including four males and four females), which on average was significantly elevated to 213% of normal in later stage sepsis caused by *ST* infection, 141% of normal in early sepsis caused by *MR* infection, and decreased to 87% of normal at the late sepsis timepoint caused by *SA* infection, and (D) α-fucosidase activity (*n* = 8 per timepoint including four males and four females), which on average increased at the late sepsis timepoint to 141% in *ST*, 151% in *SA*, and 124% in *MR* infections, while decreasing to 82% of normal at the later sepsis timepoint and 84% of normal in the early sepsis timepoint among *EC* and *SA* infections, respectively. Data are presented as means ± SD. Unpaired Mann–Whitney parametric statistical significance is denoted by *P < 0.05, **P < 0.01, or ***P < 0.001.

β-galactosidase activity was significantly elevated as compared to uninfected littermates at the late sepsis timepoint by all bacterial pathogens except for *SA*, which generated higher variability in these measurements. Activity was increased in sepsis caused by all four bacterial pathogens (Figure 5A). The *MR* infection induced statistically significant increases of β-galactosidase activity during both early- and late-sepsis while *SA* infection resulted in a slight decrease during the early sepsis timepoint and a delayed trend toward elevation at the late sepsis timepoint.

β-N-acetylglucosaminidase activity levels were statistically unchanged in early sepsis timepoints regardless of pathogen, but were significantly increased in late sepsis caused by *ST, SP*, and *SA*. No changes were measured in sepsis caused by *EC* or *MR* at either postinfection timepoint (Figure 5B).

α-Mannosidase activity was also uniquely affected in sepsis by these distinct bacterial pathogens. Activity was significantly elevated in late sepsis only by *ST* infection (Figure 5C). Activity was also increased in early sepsis caused by *MR* infection. α-Mannosidase activity decreased slightly but significantly during late sepsis caused by *SA* infection. No significant changes in α-mannosidase activity were detected in sepsis caused by *EC* or *SP* pathogens.

α-Fucosidase activity levels were also varied in sepsis caused by discrete bacterial pathogens. α-Fucosidase activity increased at the late-sepsis timepoint in *ST, SA*, and *MR* infections compared to healthy littermates. α-fucosidase activity during the *EC* infection decreased at the late-sepsis timepoint, while activity in early sepsis with *SA* infection also decreased. Similar to measurements of β-galactosidase activity, *MR* infection was the only context wherein α-fucosidase activity steadily increased during both the early- and late-sepsis timepoints compared to healthy littermates (Figure 5D).

### Glycosidase activities of bacterial pathogens

Changes in these four glycosidase activities could theoretically originate from the host and/or the pathogen. A genomic analysis of the bacterial pathogens used in eliciting experimental sepsis revealed that some had the potential to contribute one or more of these glycosidase activities. We therefore measured all four glycosidase activities in isolated cultures (10^9^ cfu/mL) of all five bacterial pathogens from the supernatant and bacterial pellet extracts in the absence and presence of the relevant glycosidase inhibitors. Activity levels were recalculated to represent 1 million bacterial cfu/mL, which is the average bacteremia level attained during the experimental sepsis protocols of this study. *EC* and *SP* genomes each encode a β-galactosidase. β-galactosidase activity was indeed detected in the supernatants and pellets of *EC* and *SP* where inhibition with DGJ was more effective towards the *EC* enzyme compared to *SP* (Figure S1A, Supplementary Material). *SP* and *SA* each encode an endo-β-N-acetylglucosaminidase, however, no activity was detected from *SA* cultures, and the *SP* enzyme was not significantly inhibited by ADN (Figure S1B, Supplementary Material). The *SP* genome encodes two α-mannosidases whereas *SA* and *ST* do not encode a known α-mannosidase. Interestingly, α-mannosidase activity was detected in the bacterial pellets, but not in the supernatant of SA, *SP*, and *ST* cultures, and this activity was not inhibited by SW (Figure S1C, Supplementary Material). The *SP* pathogen encodes an α-fucosidase while *EC* does not. α-Fucosidase activity was produced at different levels by both pathogens while DFJ did not inhibit this activity (Figure S1D, Supplementary Material). When units of activity were compared, findings indicated little if any contribution of bacterial glycosidase activities originating from the bacterial pathogens used in these experimental sepsis protocols.

### Blood plasma glycosidase activities in healthy humans and sepsis patients

Blood samples were acquired from healthy adult human volunteers (*n* = 42; 19 females and 23 males) and from consenting deidentified adult human patients admitted to hospital care with a diagnosis of severe sepsis or septic shock (*n* = 88; 34 females and 54 males). Platelet-poor plasma was prepared and analyzed as above for each of the four glycosidases. In comparing all four glycosidase activities in the plasma at pH 7.4, there were no statistically significant differences observed among healthy human males and females (Table S2, Supplementary Material). Because all sepsis patients recruited had been provided intravenous fluid therapy, thereby substantially increasing vascular volume and diluting total protein concentrations, we standardized our findings to compare human glycosidase activities in units of activity per gram of plasma protein.

**Fig. 6.**
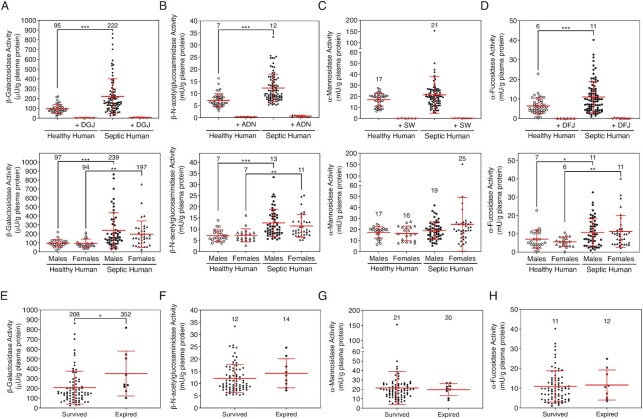
Glycosidase activities measured at pH 7.4 in healthy and septic humans. (A)–(D) Glycosidase enzyme activities indicated were assayed identically to the murine studies among individual healthy humans (*n* = 42) and human patients diagnosed with sepsis (*n* = 88). A fraction of samples (*n* = 6 for each glycosidase activity) were further compared in the presence of the relevant glycosidase inhibitor as indicated. Activities were further compared among males and females and all activities are presented using µU or mU per gram of plasma protein to control for fluid therapy provided to all septic patients prior to blood draws. A unit was defined as 1 μmole of substrate hydrolyzed per min. Average activities are denoted with numbers above the dot plots. (E) and (F) Glycosidase activities plotted among patients whom either survived or expired during sepsis are plotted and compared. Data are presented as means ± SD. Unpaired Mann–Whitney nonparametric statistical significance is denoted by *P < 0.05, **P < 0.01, or ***P < 0.001.

Compared to healthy human volunteers, β-galactosidase activity was significantly increased among septic patients by 233% on average with some patients showing much higher levels ranging from 4- to 9-fold elevations compared to healthy human controls and was completely inhibited by DGJ (Figure 6A). β-N-acetylglucosaminidase activity in the plasma was increased by 170% on average compared to healthy human controls with some patients showing 3-fold increases, and this activity was fully inhibited by ADN (Figure 6B). α-Mannosidase activity was not significantly affected on average in this septic patient population and was fully inhibited by SW. However, some septic patients, had α-mannosidase activity levels above the normal range by 2- to 10-fold (Figure 6C). α-Fucosidase activity was significantly increased to 183% of normal on average compared to healthy human controls and activity was completely inhibited by DFJ (Figure 6D). Moreover, the plasma of some sepsis patients contained activity levels 3- to 7-fold above normal.

Considering again the potential for microbial pathogen contribution to glycosidase activities, we calculated each glycosidase activity in the supernatant and pellets of the five bacterial pathogens at a bacterial titer of 100 cfu/mL, which more closely reflects the much lower blood cfu of bacteremia measured in human sepsis (38). Considering this low cfu/mL in human sepsis, our findings indicate that the four glycosidase activities measured in the blood of human patients during sepsis are of host origin (Figure S1, Supplementary Material).

Among the sepsis patient population included in this study, nine patients expired with disease signs including aspiration pneumonia, ruptured abdominal abscess, kidney dysfunction, urinary tract infections, pancolitis, hepatic abscess, and cellulitis. Remarkably, there was a statistically significant increase in β-galactosidase activity in the plasma of sepsis patients who expired compared to sepsis patients who survived indicating a prognostic indicator of increased likelihood of mortality (Figure 6E). There were no other prognostic indications of decreased survival in comparisons of β-N-acetylglucosaminidase, α-mannosidase, and α-fucosidase activities elevated in human sepsis (Figure 6F–H).

## Discussion

Establishing the origins and normal ranges of four major glycosidase activities among males and females in blood plasma and sera of mice and humans at physiological pH 7.4 has thereby further revealed significant excursions of these enzyme activities in sepsis. Previously published measurements of β-galactosidase, β-N-acetyl-glucosaminidase, α-mannosidase, and α-fucosidase activities in the blood have used experimentally acidified assay conditions reflecting their low pH optima (pH 4.3 to 5.5) to achieve highest assay sensitivities. Our previous studies indicated that these glycosidases may be active at pH 7.4 and responsible for the progressive remodeling of nascent glycoproteins with increasing molecular age in the blood circulation of healthy mammals (14). Although the specific activities of all four glycosidases studied herein were substantially reduced compared to values obtained at their low pH optima, we found that significant enzyme activity remained averaging between 3% and 7% at pH 7.4. Our findings herein have also identified the involvement of mammalian glycosidase genes responsible for a significant portion or all of the enzyme activity detected. The presence of normal ranges of multiple glycosidase activities in healthy uninfected mammalian blood plasma may be attributed to a basal level of lysosomal secretion or normal cell breakdown, while infection, inflammation, and traumatic injury may be responsible for increasing glycosidase release above basal levels in the onset of disease. Specific activities were significantly higher in sera versus plasma for all glycosidases except fucosidase, consistent with reports of platelets as stores of glycosidases that can be released into the bloodstream (12, 39, 40). Excursions of glycosidase activities outside of the normal ranges were common in both mouse and human sepsis and included elevated β-galactosidase activity as a prognostic indication of increased likelihood of death among human sepsis patients.

Elevations of blood glycosidases measured in experimentally acidified serum assay conditions have been previously linked to alcohol abuse, arthritis, cancer, hypertension, Lowe syndrome, myocardial infarction, and sepsis (13, 15, 41–47). In addition, defects in mannose-6-phosphate-dependent lysosomal trafficking result in the increased secretion of multiple glycosidases into the bloodstream (48, 49). In the bloodstream, glycosidases appear to be involved in the stepwise exoglycosidic remodeling of blood glycoproteins with increased glycoprotein age thereby exposing cryptic ligands of endocytic lectin receptors of which the Ashwell–Morell receptor is the prototypical example (14, 50). Moreover, this mechanism can determine glycoprotein half-life in concert with a family of lectin receptors that each binds a repertoire of glycoprotein ligands present among remodeled glycan linkages. Our demonstration that all four glycosidase activities assayed retain significant activity in plasma and sera at pH 7.4 supports other findings that secreted glycosidases can alter the structure and function of glycoproteins that make up the majority and diversity of the blood proteome. Indeed, we have found that this mechanism is involved in Gram-negative bacterial infections with the induction of host neuraminidases that target for rapid clearance the anti-inflammatory alkaline phosphatase isozymes in determining the severity of inflammation and the frequency of death in experimental sepsis (15). Alkaline phosphatase augmentation is among current human clinical trials for the treatment of sepsis (51).

β-galactosidase activity at pH 7.4 is identical among murine males and females in platelet-poor plasma while levels are 10- to 20-fold higher in sera again indicating sources such as platelets as responsible for this increase (52). *Glb1*-null mice were deficient of β-galactosidase activity by 80% to 90% in both plasma and sera. Females generally had lower levels in sera, but not plasma, suggesting a distinct isozyme may be released from platelets at higher levels in males. One candidate is the mammalian Galc galactosylceraminidase. Similarly, β-N-acetylglucosaminidase activity is elevated in sera compared to plasma, further implicating the alpha-granules of platelets (39, 40). Unlike β-galactosidase activity, β-N-acetylglucosaminidase activity is significantly higher in healthy normal murine females as compared to males. In both sexes, this activity is highly dependent upon the *HexB* gene in plasma, as is more than 90% of activity in sera. Remaining β-N-acetylglucosaminidase activity in HexB deficiency may reflect other isozymes including HexC and HexD (53). α-Mannosidase activity is normally lower among healthy murine females compared to males while this activity is also elevated approximately 2-fold in sera compared to plasma. In the blood, α-mannosidase activity is a combination of two or more isoyzmes as Man2a1 (α-mannosidase-II) contributed only 50% of measured activity. Our findings indicate that Man2a1 is secreted from its membrane-bound position in the medial Golgi. α-Fucosidase activity was simpler in analysis, being identical among murine males and females and unchanged comparing plasma or sera. Interestingly, all measurable α-fucosidase activity was dependent upon the Fuca1 fucosidase indicating that Fuca2 does not contribute independently to fucosidase activity in our assays of plasma and sera. In that regard, it is worth noting that our assays of specific activities and their inhibition use synthetic substrates that may not substitute for physiological substrates among blood glycoproteins.

Excursions of glycosidase activity levels beyond normal ranges in sepsis was common to both mouse and human species. Among the pathogens studied, bacterial glycosidase activities do not appear to contribute significantly at bacterial cfu concentrations attained in the blood during sepsis among both species. This does not eliminate the possibility that cellular infection and host pathogenesis among tissues includes the action of bacterial glycosidases, such as indicated with *S. pneumoniae* infections (54). In fact, bacterial glycosidase-mediated glycocalyx degradation may contribute to the onset of various human diseases (55, 56). in our studies, host glycosidases are most greatly affected by *Salmonella* infections which led to the largest increases measured among all four glycosidase activities. This may reflect in part the oral route of *ST* infection used in our studies and *ST* tissue colonization prior to the onset of bacteremia. Among the other four bacterial pathogens tested, glycosidase activity excursions were mostly increases, with some activities decreased from normal for reasons currently unknown. In human sepsis, in which pathogens involved are more diverse and often remain unidentified, there was a significant elevation in all glycosidase activities except for α-mannosidase activity, which was not significantly different from healthy human subjects. However, α-mannosidase activity in healthy humans appeared to segregate into two groups with activity differences of about 2-fold. This may reflect the reported presence of α-mannosidase gene mutations in humans (8, 29), and which may obscure the presence of significant excursions in α-mannosidase activity among individual sepsis patients.

The constituitive and regulated activities of these glycosidases measured at pH 7.4 in the plasma and sera of mice and humans reflect in part an ongoing process previously linked to the half-lives and endocytic turnover of secreted and cell surface glycoproteins (14, 15, 57). As those studies demonstrated, even relatively small changes in the levels of these hydrolases can have rapid and consequential impacts on the abundance, and thereby, function of key physiological regulators. The largest changes in glycosidase activities were generally seen in the *ST* infections by oral inoculation. Whether this reflects *ST* itself or longer periods of disease progression with pathogen colonization and damage to tissues and organs is not yet known. Among human glycosidase activity excursions in sepsis, a significant elevation of β-galactosidase activity was linked to an increased likelihood of patient death. β-galactosidase is also known as a biomarker of cellular senescence in multiple cell types (58). In sepsis, kidney damage leading to renal failure is common and associated with increased β-galactosidase activity in serum and urine, reflecting a worsening of the disease state (59). Our analyses together indicate an association of high β-galactosidase activities with kidney disease and patient death. Further analyses of these and additional septic patients will be required to identify whether β-galactosidase activity has an independent association with patient death. These studies together comparing both mouse and human species have established normal ranges of glycosidase activity levels at physiological pH 7.4 in the bloodstream, and have documented activity excursions in sepsis that may modulate the blood proteome in pathogenesis and thereby influence disease outcome.

## Methods

### Mice and sample acquisition

All mouse strains used were backcrossed at least six generations into the C57BL/6 J strain background and studied with wild-type littermates at 10 to 12 weeks of age, except for the *Fuca1*-null mouse strain (36), which were 20 to 22 weeks old at the time of experimentation. Mice lacking functional genes *Glb1, HexB*, or *Man2a1* were previously described (24, 26, 30). Animals were fully anesthetized within an isoflurane chamber. Murine blood was collected via cardiac puncture using a 27-gauge half inch needle attached to a 1-mL syringe. Glycosidase activity can be affected by poor technique of sample collection and the anticoagulants present. Cardiac punctures obtain a large sample with minimal trauma and samples with pink or red hemolysis were discarded from the study. Whole blood was added to serum separator tubes (SST) and EDTA-coated collection tubes. Serum was allowed to coagulate for a minimum of 30 min and processed before 60 min of room temperature standing time. Serum was isolated by centrifugation of SSTs at 3,000 rcf at 20°C for 10 min. Platelet poor plasma was isolated by centrifugation of EDTA tubes at 3,000 rcf at 20°C for 10 min, twice, and harvesting the supernatant. Samples were flash frozen in a 1:1 mixture of ethanol and dry ice and stored at −80°C.

### Human sepsis patient recruitment and blood acquisition

Adult patients were recruited upon informed consent by Santa Barbara Cottage Hospital (SBCH) Research Coordinators under IRB #16–72 u if they met the “Sepsis-2” diagnosis criteria (60). Anticoagulant-treated (citrated) human blood was collected and processed within 2 hours. Whole blood was centrifuged at 3,000 rcf at 20°C for 12 mins. Aliquots of platelet-poor plasma were flash frozen in a 1:1 mixture of ethanol and dry ice and stored at −80°C prior to analysis.

### Healthy human blood samples

Blood was obtained from consenting healthy donors via certified phlebotomists at the SBCH affiliate Pacific Diagnostics Laboratory and as part of IRB #16–72 u. Anticoagulant-treated (citrated) whole blood was centrifuged at 3,000 rcf at 20°C for 12 mins, twice. The resulting platelet-poor plasma supernatants were flash frozen in a 1:1 mixture of EtOH and dry ice, and stored at −80°C.

### Bacterial pathogens

Experimental sepsis was induced using the following bacterial pathogens *S. enterica* Typhimurium strain 14028 (*ST*), *E. coli* strain ATCC 25922 (*EC*), *S. pneumoniae* strain D39 (*SP*), *S. aureus* strain USA300 MRSA (*MR*), and *S. aureus* strain Newman MSSA (*SA*). Infection protocols were undertaken as previously reported (15).

### β-Galactosidase assay

β-Galactosidase activity was measured using the substrate 4-methylumbelliferyl-β-D-galactopyranoside (MUGal) purchased from Sigma-Aldrich (Sigma, M1633). Plasma or sera samples (10 μL) were added to 80 μL of assay buffer consisting of 50 mM Tris-HCl (pH7.4; Thermo-Fisher BP152-5), or 50 mM sodium acetate (pH 4.3; Thermo-Fisher, S209) with addition of 10 μL of 0.875 mM MUGal in the relevant buffer. To measure inhibition of β-galactosidase activity, 10 μL of 10 mM N-(n-nonyl)-DGJ (Santa Cruz Biotechnology, SC-221975) in 100% DMSO was added to 70 μL of the relevant assay buffer prior to addition of sample and substrate. The 100 μL assay mixtures were incubated for 60 min at 37°C and the reactions were stopped with the addition of 200 μL of 0.2 M Glycine–NaOH at pH 10.8 (Sigma, G2879). Measurements of activity were determined by the amounts of 4MU detected at 445 nm (ex/360 nm) with a SpectraMax iD3 using the 3 × 3 well scan setting. Serial dilutions of 4MU (Sigma, M1508) were used in all glycosidase assays to produce a standardized specific activity curve. A unit of activity was defined as 1 μmole of substrate hydrolyzed per minute. Purified β-galactosidase enzyme purchased from New England Biolabs (P0746S/L) served as a positive control.

### β-N-acetylglucosaminidase assay

β-N-acetylglucosaminidase activity was measured using the substrate 4-methylumbelliferyl-N-acetyl-β-D-glucosaminide (MUG) purchased from Sigma-Aldrich (Sigma, 69,585). Plasma or sera samples (10 μL) were added to 80 μL of assay buffer consisting of 50 mM Tris buffer (pH 7.4), or were diluted 1:10 in PBS (Gibco 14190–144) and added to 80 μL of 50 mM phosphate–citrate buffer (pH 4.7; Sigma, P4809) with the addition of 3.75 mM MUG in 10 μL of the relevant buffer. To measure inhibition of β-N-acetylglucosaminidase activity, 10 μL of 2.5 mM ADN (Sigma, 90921) in 100% DMSO was added to 70 μL of the relevant assay buffer prior to addition of sample and substrate. The 100 μL assay mixtures were incubated for 30 min at 37°C and the reactions were stopped with the addition of 200 μL of 0.2 M Glycine–NaOH (pH10.8; Sigma, G2879). Measurements of activity were determined by the amounts of 4MU detected at 445 nm (ex/360 nm) with a SpectraMax iD3 using the 3 × 3 well scan setting. A unit of activity was defined as 1 μmole of substrate hydrolyzed per minute. Purified β-N-acetylglucosaminidase enzyme purchased from New England Biolabs (P0744S/L) served as a positive control.

### α-Mannosidase assay

α-Mannosidase activity was measured using the substrate 4-methylumbelliferyl α-D-mannopyranoside (MUM) purchased from Sigma-Aldrich (Sigma M3657). Plasma or sera samples (10 μL) were added to 80 μL of assay buffer consisting of 50 mM Tris buffer (pH 7.4), or were diluted 1:10 in PBS (Gibco 14190–144) and added to 80 μL of 50 mM sodium acetate buffer (pH 4.3; Thermo-Fisher, S209) with the addition of 3.75 mM MUM in 10 μL of the relevant buffer. To measure inhibition of α-mannosidase activity, 10 μL of 2.5 mM SW (Santa Cruz Biotechnology, SC-201362) in 100% DMSO was added to 70 μL of the relevant assay buffer prior to addition of sample and substrate. The 100 μL assay mixtures were incubated for 60 min at 37°C and the reactions were stopped with 200 μL of 0.2 M Glycine–NaOH (pH 10.8; Sigma, G2879). Measurements of activity were determined by the amounts of 4MU detected at 445 nm (ex/360 nm) with a SpectraMax iD3 using the 3 × 3 well scan setting. A unit of activity was defined as 1 μmole of substrate hydrolyzed per minute. Purified α-mannosidase enzyme purchased from New England Biolabs (P0768S/L) served as a positive control.

### α-Fucosidase assay

α-Fucosidase activity was measured using the substrate 4-methylumbelliferyl α-L-fucopyranoside (MUF) purchased from Sigma-Aldrich (Sigma, M3657). Plasma or sera samples (10 μL) were added to 80 μL of assay buffer consisting of 50 mM Tris-HCl (pH 7.4), or were diluted 1:10 in PBS (Gibco, 14190–144) and added to 80 μL of 50 mM phosphate–citrate buffer (pH 5.5; Thermo-Fisher, P4809) with the addition of 0.625 mM MUF in 10 μL of the relevant buffer. To measure inhibition of α-fucosidase activity, 10 μL of 2.5 mM DFJ (Santa Cruz Biotechnology, SC-205644) in 100% DMSO was added to 70 μL of the relevant assay buffer prior to addition of sample and substrate. The 100 μL assay mixtures were incubated for 60 min at 37°C and the reactions were stopped with 200 μL of 0.2 M Glycine–NaOH (pH 10.8; Sigma, G2879). Measurements of activity were determined by the amounts of 4MU detected at 445 nm (ex/360 nm) with a SpectraMax iD3 using the 3 × 3 well scan setting. A unit of activity was defined as 1 μmole of substrate hydrolyzed per minute. Purified α-fucosidase enzyme purchased from New England Biolabs (P0748S/L) served as a positive control.

### Bacterial culture and glycosidase activity

Bacterial pathogens were cultured in vitro and assayed for glycosidase activity. Triplicate measurements were made of bacterial pellets and supernatant samples from overnight cultures prepared as follows. *Salmonella enterica Typhimurium* strain 14028 (*ST*) was streaked from frozen stocks onto Luria–Bertani (LB) agar plates and incubated overnight at 37°C. Single colonies were inoculated into LB broth and incubated overnight with shaking at 37°C. *Escherichia coli* strain ATCC 25922 (*EC*) was streaked from frozen stocks onto tryptic soy (TS) agar plates and incubated overnight at 37°C. Single colonies were inoculated into TS broth and incubated overnight with shaking at 37°C. *Streptococcus pneumoniae* D39 (*SP*) was streaked from frozen stocks onto Todd–Hewitt (TH) broth agar plates containing 2% yeast extract and incubated overnight at 37°C in a 5% CO_2_ incubator. Single colonies were inoculated into Todd–Hewitt broth containing 2% yeast extract and incubated overnight without shaking at 37°C in a 5% CO_2_ incubator. After overnight incubation, bacteria were reinoculated as a 1:10 subculture into fresh TH broth and cultured to mid-log phase (A600 = 0.4). *Staphylococcus aureus* strain USA300 MRSA (*MR*) and *S. aureus* strain Newman MSSA (*SA*) were streaked from frozen stocks onto TS agar plates and incubated overnight at 37°C. Single colonies were inoculated into TS broth and incubated overnight with shaking at 37°C. After overnight incubation, bacteria were reinoculated as a 1:100 subculture into fresh TS broth and cultured to mid-log phase (A600 = 0.4). Bacterial cell pellets and the culture supernatants were then collected for glycosidase activity analyses at pH 7.4. All bacteria were pelleted by centrifugation at 1,500 × *g* for 5 min, washed twice in 1 mL PBS (Gibco 14190–144), resuspended in 200 µL PBS at a concentration of 1 × 10^9^ cfu/mL, and flash frozen. Bacterial cell supernatants from the first centrifugation were collected in 200 µL aliquots and flash frozen. Activities were further calculated by mathematical dilution at 1 × 10^6^ and 1 × 10^2^ cfu/mL.

### Bacterial genome analysis

*Salmonella enterica Typhimurium* str. 14028 was queried for the four glycosidase enzymes assayed using NCBI’s Genome Assembly ID 382525 (https://www.ncbi.nlm.nih.gov/genome/proteins/152?genome_assembly_id=382525). *Escherichia coli* strain ATCC 25922 was queried for the four glycosidase enzymes assayed using NCBI’s Genome Assembly ID 163019 (https://www.ncbi.nlm.nih.gov/genome/proteins/167?genome_assembly_id=163019; Locus Tag: K758_RS07430, Assembly 163019). *Streptococcus pneumoniae* D39 was queried for the four glycosidase enzymes assayed using NCBI’s Genome Assembly ID 375262 (https://www.ncbi.nlm.nih.gov/genome/proteins/176?genome_assembly_id=375262; Locus Tag: SPD_RS00330, Assembly 375262; Locus Tag: SPD_RS04590, Assembly 375262; Locus Tag: SPD_RS10430, Assembly 375262; Locus Tag: SPD_RS10440, Assembly 375262; and Locus Tag: SPD_RS10545, Assembly 375262). *Staphylococcus aureus* strain Newman MSSA was queried for the four glycosidase enzymes assayed using NCBI’s Genome Assembly ID 299277 (https://www.ncbi.nlm.nih.gov/genome/proteins/154?genome_assembly_id=299277; Locus Tag: NWMN_RS05165, Assembly 299277). *Staphylococcus aureus* strain USA300 MRSA was queried for the four glycosidase enzymes assayed using NCBI’s Genome Assembly ID 366661 (https://www.ncbi.nlm.nih.gov/genome/proteins/154?genome_assembly_id=366661).

### Statistical analysis

All data were analyzed as mean ± SD. unless otherwise indicated. Unpaired nonparametric Mann–Whitney statistical test with GraphPad Prism software (Version 8.0) was used to determine statistical significance. *P*-values of less than 0.05 were considered significant. Statistical significance was denoted by *P < 0.05, **P < 0.01, or ***P < 0.001.

## Supplementary Material

pgac113_Supplemental_Files

## Data Availability

All data is included in the manuscript and/or supporting information.
