# Correction to: Establishment of blood glycosidase activities and their excursions in sepsis

**DOI:** 10.1093/pnasnexus/pgad463

**Published:** 2024-01-30

This is a correction to: Benjamin S Haslund-Gourley, Peter V Aziz, Douglas M Heithoff, Damien Restagno, Jeffrey C Fried, Mai-Britt Ilse, Hannah Bäumges, Michael J Mahan, Torben Lübke, Jamey D Marth, Establishment of blood glycosidase activities and their excursions in sepsis, *PNAS Nexus*, Volume 1, Issue 3, July 2022, pgac113, https://doi.org/10.1093/pnasnexus/pgac113

During a retroactive audit conducted by *PNAS Nexus*, it was discovered that this paper was missing a statement acknowledging compliance with the *PNAS Nexus* Human and Animal Participants and Clinical Trials policy:

All experiments involving human participants and their recruitment were approved by the IRB committees of the University of California Santa Barbara, the University of California San Diego, and the SBP Medical Discovery Institute. All participants provided consent in writing for blood draws and urine collection. The IACUC of the SBP Medical Discovery Institute approved all animal (mouse) studies undertaken in this research.

This error has been corrected in the original article.

