# Correction to: Establishment of blood glycosidase activities and their excursions in sepsis

**DOI:** 10.1093/pnasnexus/pgaf265

**Published:** 2025-08-26

This is a correction to: Benjamin S Haslund-Gourley, Peter V Aziz, Douglas M Heithoff, Damien Restagno, Jeffrey C Fried, Mai-Britt Ilse, Hannah Bäumges, Michael J Mahan, Torben Lübke, Jamey D Marth, Establishment of blood glycosidase activities and their excursions in sepsis, *PNAS Nexus*, Volume 1, Issue 3, July 2022, pgac113, https://doi.org/10.1093/pnasnexus/pgac113

The originally published version of this manuscript was found by the authors to contain the following data processing errors.

During data analysis in composing Figure 6, it was recently noted that close to half of the plasma sample measurements were mistakenly calculated as 30 minute incubations, even though all plasma samples were incubated for 60 minutes as per Methods. Therefore, those data points were re-analyzed and re-plotted correctly in the revised Table S2 and Figure 6. These corrections have minimal impact but now demonstrate that plasma alpha-mannosidase activity is also significantly induced in human sepsis.

The revised versions of Table S2 and Figure 6, incorporating the corrected values, are provided below:
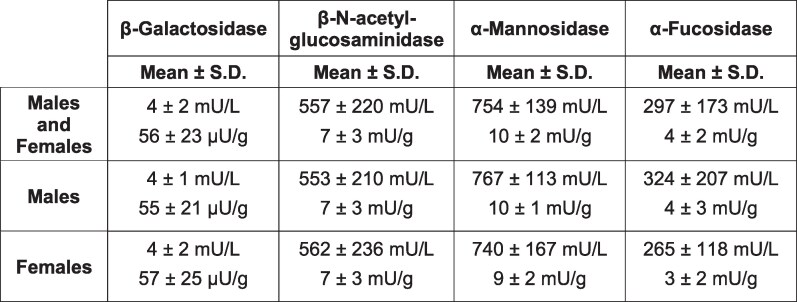


**Figure:**
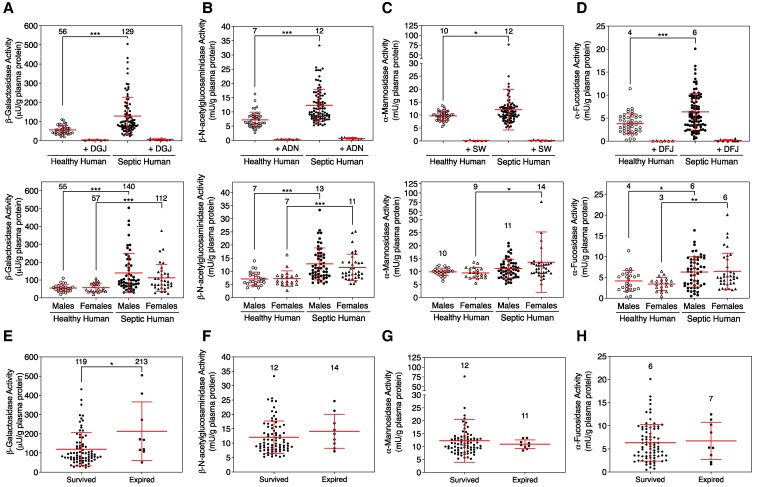


Additionally, on page 7 of the article, in the subsection entitled ‘Blood plasma glycosidase activities in healthy humans and sepsis patients’, the following text requires correction:

‘α-Mannosidase activity was not significantly affected on average in this septic patient population and was fully inhibited by SW. However, some septic patients, had α-mannosidase activity levels above the normal range by 2- to 10-fold (Figure 6C).

This text should instead read:

‘α-Mannosidase activity was also significantly affected in this septic patient population and was fully inhibited by SW. However, some septic patients, had α-mannosidase activity levels above the normal range by 2- to 6-fold (Figure 6C).’

On page 8 of the article, in the Discussion section, the following text requires correction:

‘However, α-mannosidase activity in healthy humans appeared to segregate into two groups with activity differences of about 2-fold. This may reflect the reported presence of α-mannosidase gene mutations in humans (8, 29), and which may obscure the presence of significant excursions in α-mannosidase activity among individual sepsis patients.’

This text should instead read:

‘However, α-mannosidase activity in humans may reflect the reported presence of α-mannosidase gene mutations in humans (8, 29), which may obscure larger excursions in α-mannosidase activity among individual sepsis patients.”

These details have been corrected only in this correction notice to preserve the published version of record.

