# Supplementary material for: Establishment of blood glycosidase activities and their excursions in sepsis
Source: PNAS Nexus. 2022 Jul 11;1(3):pgac113. doi: 10.1093/pnasnexus/pgac113 (PMC9364217; doi:10.1093/pnasnexus/pgac113)
Supplement: pgac113_Supplemental_Files [file pgac113_supplemental_files.zip › PNASNEXUS-PNASNEXUS-2022-00198-T-s01.docx]

**Supplemental Tables and Figure Legend**

| **Table S1. Healthy murine glycosidase activities at pH 7.4 in platelet poor plasma and serum.** | | | | | |
| --- | --- | --- | --- | --- | --- |
|  |  | **β-Galactosidase (mU/L)** | **β-N-acetyl- glucosaminidase (mU/L)** | **α-Mannosidase (mU/L)** | **α-Fucosidase (mU/L)** |
|  |  | **Mean ± S.D.** | **Mean ± S.D.** | **Mean ± S.D.** | **Mean ± S.D.** |
| **Males  and  Females** | **Plasma** | 15 ± 3 | 2,863 ± 895 | 2,344 ± 400 | 266 ± 48 |
|  | **Serum** | 156 ± 65 | 3,614 ± 799 | 2,401 ± 371 | 275 ± 61 |
| **Males** | **Plasma** | 16 ± 2 | 2,012 ± 347 | 2,596 ± 372 | 280 ± 50 |
|  | **Serum** | 195 ± 70 | 3,185 ± 534 | 2,645 ± 315 | 290 ± 68 |
| **Females** | **Plasma** | 14 ± 3 | 3,525 ± 535 | 2,092 ± 242 | 245 ± 41 |
|  | **Serum** | 118 ± 29 | 3,948 ± 834 | 2,156 ± 240 | 253 ± 46 |

| **Table S2. Healthy human glycosidase activities at pH 7.4 in platelet poor plasma.** | | | | |
| --- | --- | --- | --- | --- |
|  | **β-Galactosidase** | **β-N-acetyl-glucosaminidase** | **α-Mannosidase** | **α-Fucosidase** |
|  | **Mean ± S.D.** | **Mean ± S.D.** | **Mean ± S.D.** | **Mean ± S.D.** |
| **Males and Females** | 7 ± 3 mU/L | 557 ± 220 mU/L | 1,329 ± 471 mU/L | 506 ± 341 mU/L |
|  | 95 ± 45 µU/g | 7 ± 3 mU/g | 17 ± 6 mU/g | 6 ± 4 mU/g |
| **Males** | 8 ± 3 mU/L | 553 ± 210 mU/L | 1,359 ± 419 mU/L | 559 ± 413 mU/L |
|  | 97 ± 42 µU/g | 7 ± 3 mU/g | 17 ± 5 mU/g | 7 ± 5 mU/g |
| **Females** | 7 ± 4 mU/L | 562 ± 236 mU/L | 1,293 ± 537 mU/L | 443 ± 223 mU/L |
|  | 94 ± 48 µU/g | 7 ± 3 mU/g | 16 ± 7 mU/g | 6 ± 3 mU/g |

**Supplemental Figure Legend.**

**Figure S1. Bacterial pathogen glycosidase activities at bacteremia levels typical of mouse and human sepsis.** (A) β-galactosidase activity, (B) β-N-acetylglucosaminidase activity, (C) α-mannosidase activity, and (D) α-fucosidase activity assayed at pH 7.4 in bacterial supernatant and pellets from overnight cultures calculated at 10^6^ and 10^2^ bacterial cfu/mL to replicate bacteremia in mice and humans, respectively. Specific activity inhibition was calculated using DGJ (C*_f_* = 1 mM), ADN (C*_f_* = 0.25 mM), SW (C*_f_* = 0.25 mM), and DFJ (C*_f_* = 0.2 mM). A unit of activity was defined as 1 μmole of substrate hydrolyzed per minute. Data (n=3) are presented as means ± S.D.
