# Supplementary figures and images for: Establishment of blood glycosidase activities and their excursions in sepsis
Source: PNAS Nexus. 2022 Jul 11;1(3):pgac113. doi: 10.1093/pnasnexus/pgac113 (PMC9364217; doi:10.1093/pnasnexus/pgac113)

Figure S1

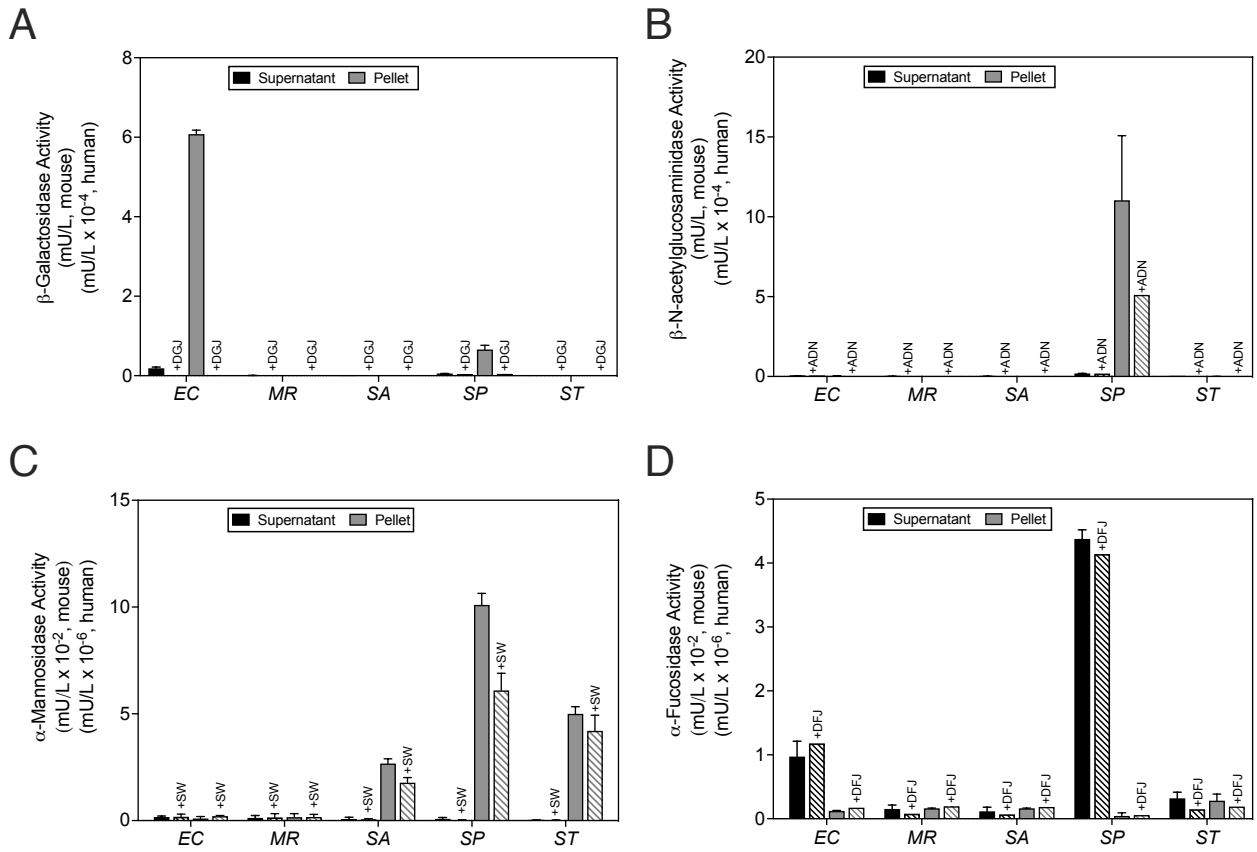

Supplement: pgac113_Supplemental_Files [file pgac113_supplemental_files.zip › PNASNEXUS-PNASNEXUS-2022-00198-T-s02.pdf]
